# New Insights in Preterm Nutrition

**DOI:** 10.3390/nu12061857

**Published:** 2020-06-22

**Authors:** Paola Roggero, Nadia Liotto, Camilla Menis, Fabio Mosca

**Affiliations:** 1 Neonatal Intensive Care Unit, Fondazione IRCCS Ca’ Granda Ospedale Maggiore Policlinico, 20122 Milan, Italy; nadia.liotto@policlinico.mi.it (N.L.); camilla.menis@gmail.com (C.M.); fabio.mosca@unimi.it (F.M.); 2 Department of Clinical Sciences and Community Health, University of Milan, 20122 Milan, Italy

Nutrition of preterm infants has a crucial role in the promotion of organ’s optimal growth and development [1,2,3,4].

In recent years, much has been researched, discussed and written on the ideal nutritional approach, suitable growth and the possible short- and long-term health consequences related to over- or undernutrition and inappropriate growth [5].

Sir David Cuthbertson [6] emphasized that “we all owe our foetal life till parturition to the passage of the nutrients we require from the blood vessels of our mothers into our blood vessels as they traverse the chorionic villi in a close relation”. Therefore, “intravenous feeding” is crucial in preterm infants who need parenteral nutrition for sustaining postnatal life, as “intrauterine feeding” ends abruptly with the clamping of the umbilical cord at birth. Preterm birth interrupts the physiological growth path of the foetus that occurs during the third trimester of pregnancy. At this stage, it is known that the foetus has a rapid growth phase that can hardly be maintained by the preterm infant due to changes in the environment related to birth. The environment to which preterm infants are exposed is very different from the maternal uterus and causes an increase in their energy expenditure due to the need to maintain thermal and metabolic homeostasis. Based on this scenario, it is crucial to define the nutritional management (in terms of intakes, timing and modalities) of preterm infants to ensure their adequate growth and development.

Important results have been achieved with regard to nutritional intake, both parenterally and enterally, in relation to gestational age and birth weight [7,8,9]. Based on the nutritional intake of the foetus in the womb, a consensus has been reached in recent decades regarding the need to guarantee “aggressive nutrition” to the preterm infant to mimic intrauterine growth and quality of growth and improve short- and long-term health outcomes [10]. Over the years, it has been observed that, despite an aggressive nutritional approach, neonatal growth did not reflect what was expected of a foetus of the same gestational age. Some authors [11] have investigated the possible causes of this postnatal growth retardation and have shown that, by analysing the actual cumulative nutrients administered, it was not always possible to achieve what was recommended due to the complexity of the daily management of preterm infants, especially if extremely preterm, due to the need to use nutritional access for other therapies. Therefore, it has been hypothesized that it may be inappropriate to compare preterm infant growth with the growth of their in utero counterparts. In this direction, the Intergrowth 21st study was designed, which suggested that the correct comparator for assessing the growth of preterm infants was a cohort of healthy preterm infants [12,13,14,15].

With this goal, the researchers of the Intergrowth 21st Consortium published preterm postnatal growth standards that allow to assess the growth of preterm infants until 64 weeks gestational age (6 months corrected age), the time at which they overlap, without the need for any adjustment, with the World Health Organization Child Growth Standards for term newborns [15]. In particular, this new approach for monitoring postnatal growth is more adequate in assessing the growth rates of preterm infants. In addition, this approach can consider “adequate” instead of “aggressive” nutritional management of preterm infants.

The timing of nutritional intervention has been also defined. Indeed, since, with the premature birth, these infants interrupt their growth trajectory and they find themselves in a nutritional emergency. Therefore, any postnatal delay in undertaking an adequate nutritional intake creates a growth restriction over time. Consequently, there is a general consensus in early nutrition immediately after birth [16,17].

Another issue that is scarcely discussed and difficult to study due to the complexity of the sample size necessary to achieve adequate study power, is related to the qualitative composition of the amino acid infusion mixtures used for parenteral nutrition of the premature infant. To date, there are no specific intravenous mixtures for preterm infants on the market, as the quality and quantity of the individual amino acids necessary for premature growth are unknown. Consequently, some proteins cannot be synthesized due to the lack of the quantity and quality of the amino acids necessary for preterm infants [18,19].

The transition phase from parenteral nutrition to enteral nutrition represents a challenge for neonatologists. This is a very vulnerable period, since the absorption of various nutrients from the intestinal lumen to the splanchnic district varies according to the type of amino acids. Therefore, it is crucial to carefully perform this transition to ensure adequate intake, mainly of protein, and consequently achieve adequate growth [20,21,22].

Regardless of parenteral nutrition, which is crucial for preterm infants in the first period of life, an increasingly central role of human milk is recognized not only for its nutritional value in terms of growth and quality of growth but also for its immune protection and for its role in modulating the microbiota, which plays a decisive role in the intestinal maturation of a highly vulnerable population such as severely preterm infants. Specifically, the metabolic capacity of the preterm gut microbiota may contribute to the production of energy and metabolites that influence gut maturation and metabolism [23,24,25,26].

Although human milk allows for the intake of functional nutrients that formulated milk cannot completely supply, it is unable to provide the protein/energy needs for preterm infants. For this reason, there is unanimous consensus on the need to fortify human milk, especially if donated. The fortification of human milk, as a supplement of macronutrients and micronutrients of bovine origin according to standard modalities, has evolved in recent decades due to the ever deeper knowledge of human milk composition and the need to guarantee the amounts of nutrients required by each infant. Indeed, through the introduction of the “adjustable” fortification mode, based on the evaluation of blood urea nitrogen, or of “targeted” fortification mode, based on the analysis of the micronutrient composition of human milk, it was possible to change the scenario of the fortification of human milk, resulting in improved growth outcomes [27].

Regarding body composition, the American Academy of Pediatrics, in 1977, recommended that body composition should replicate in utero body composition. Nevertheless, preterm infants at term-corrected age have altered body composition compared to term infants. Specifically, fat body mass in preterm infants at term-corrected age is significantly higher than that of full-term newborns [28]. In contrast, recently, it has been demonstrated that with increasing energy and protein intake during the hospital stay, higher fat-free mass at discharge was achieved, and fat-free mass was positively associated with neurodevelopmental outcomes at 12 and 24 months corrected age [29].

## Future Research

The new concept of growth for preterm infants, based on growth trajectories of infants instead of foetuses, could have beneficial long-term effects on health. It would therefore be desirable that multi-centre randomized controlled trials be designed to explore the effect of early nutrition and growth on long-term health.
